# Dynamic CT angiography for cyberknife radiosurgery planning of intracranial arteriovenous malformations: a technical/feasibility report

**DOI:** 10.1515/raon-2015-0006

**Published:** 2015-03-25

**Authors:** Anoop Haridass, Jillian Maclean, Santanu Chakraborty, John Sinclair, Janos Szanto, Daniela Iancu, Shawn Malone

**Affiliations:** The Ottawa Hospital, Ottawa, Ontario, Canada

**Keywords:** arteriovenous malformation, radiosurgery, Cyberknife, dynamic CT angiogram

## Abstract

**Background.:**

Successful radiosurgery for arteriovenous malformations (AVMs) requires accurate nidus delineation in the 3D treatment planning system (TPS). The catheter biplane digital subtraction angiogram (DSA) has traditionally been the gold standard for evaluation of the AVM nidus, but its 2D nature limits its value for contouring and it cannot be imported into the Cyberknife TPS. We describe a technique for acquisition and integration of 3D dynamic CT angiograms (dCTA) into the Cyberknife TPS for intracranial AVMs and review the feasibility of using this technique in the first patient cohort.

**Patients and methods.:**

Dynamic continuous whole brain CT images were acquired in a Toshiba 320 volume CT scanner with data reconstruction every 0.5 sec. This multi-time-point acquisition enabled us to choose the CT data-set with the clearest nidus without significant enhancement of surrounding blood vessels. This was imported to the Cyberknife TPS and co-registered with planning CT and T2 MRI (2D DSA adjacent for reference). The feasibility of using dCTA was evaluated in the first thirteen patients with outcome evaluation from patient records.

**Results.:**

dCTA data was accurately co-registered in the Cyberknife TPS and appeared to assist in nidus contouring for all patients. Imaging modalities were complementary. 85% of patients had complete (6/13) or continuing partial nidus obliteration (5/13) at 37 months median follow-up.

**Conclusions.:**

dCTA is a promising imaging technique that can be successfully imported into the Cyberknife TPS and appears to assist in radiosurgery nidus definition. Further study to validate its role is warranted.

## Introduction

Intracranial arteriovenous malformations (AVM) are congenital vascular abnormalities present in approximately 0.01–0.5% of the population.^1^ In AVM, arteries supply a serpiginous collection of vessels, called the nidus, which shunt blood from the feeding arteries directly to the draining veins without an intervening capillary bed. The nidus and draining veins become enlarged and tortuous to cope with the increased flow. Intracranial AVMs are clinically important as there is 1–5% annual risk of haemorrhage.^2^,^3^

Advances in the treatment of AVMs have resulted in a decrease in associated morbidity and mortality in the last two decades.^4^ Although microvascular surgery remains the gold standard treatment, stereotactic radiosurgery (RS) is proving increasingly useful in the treatment of small inoperable deep seated AVMs, those in eloquent areas where the risk of surgical morbidity is high, in medically inoperable patients and as part of a multi-therapy approach for larger complex AVMs.^5^–^7^

The goal of RS for AVMs is to treat the nidus to a high dose while simultaneously minimising dose to the surrounding normal tissue. Improvements in image guidance, computing and radiation delivery technology in the last two decades have made it feasible to deliver accurate highly conformal RS plans. However, a successful outcome following RS - optimal AVM obliteration with minimal toxicity - is dependent on accurate definition of the nidus. This has always been challenging and inaccuracies in nidus definition are an important cause for treatment failure.^8^,^9^

The gold standard for imaging vascular structures, such as the AVM nidus, is the catheter biplane high resolution digital subtraction angio-gram (DSA). The excellent temporal resolution of the DSA allows differentiation of the nidus from the feeding arteries and draining veins. However, the 2D nature of DSA images means they are of limited use to contour the nidus in 3D RS planning. Furthermore, DSA images cannot be co-registered in the Cyberknife (CK) RS treatment planning system as stereotactic localisation is frame-based in DSA, whereas CKRS uses skull tracking. Therefore, 3D imaging modalities, such as CT angiograms (CTA) and MR angiograms (MRA) are used for RS planning. Whilst CTA and MRA provide a view of the vascular tree with excellent 3D localization, the drawback of the standard ‘static’ CTA/MRA is that the images represent a snapshot of the blood flow through the AVM and draining veins at a pre-determined acquisition time. This snapshot is unlikely to be the optimal time-point for viewing the nidus, which can make differentiation of the nidus from the surrounding angio-architecture challenging.

Dynamic CT angiography (dCTA) is a non-invasive vascular imaging technique that has been shown to provide both high temporal and spatial resolution in a 3D volume dataset. The entire cerebral volume can be imaged in a single rotation of the gantry, generating a full CT dataset of the cerebral vasculature every second. The scanner splits each dataset to generate whole brain CTA volume with temporal resolution of 0.5 sec. The utility of dCTA has been described in the evaluation of AVMs^10^,^11^, but there are no published reports of using it to aid RS planning. Potentially dCTA would allow delineation of the nidus on the CT dataset captured at the point when the nidus is clearest. We incorporated dCTA into our CKRS planning protocol in 2010 and present a feasibility report regarding our initial findings in the first cohort of patients.

## Patients and methods

All patients with AVMs who were treated with CKRS at our hospital between October 2010 and April 2012 underwent a dynamic CT angiogram as part of the planning process. Written informed consent of patients was obtained for the treatments and for the scientific use of the clinical data according to Declaration of Helsinki.

### Procedure for dCTA

Patients were scanned in an Aquilion ONE multi-detector volume CT scanner (Toshiba, Medical Systems, Japan) in a supine head first position with IV access as per departmental protocol. This system is equipped with 320 ultra-high resolution detector rows (0.5mm in width), 512 × 512 matrix and images 16cm in z-axis in a single gantry rotation which covers the entire brain. Whole brain CT data is acquired at multiple time points as per dCTA protocol. From this dataset Dynamic (time resolved) CT angiography (dCTA) images were reconstructed enabling the analysis of the blood flow in the entire cranial circulation in a non-invasive way with high spatial and temporal resolution. The radiation dose for the dCTA acquisition is approximately (DLP= Dose length product) 2170 mGycm = effective dose 5 mSv, for comparison radiation dose for non-contrast CT head will be (DLP) 1335 mGycm) = 3 mSv.

### Timing bolus

Following acquisition of a scout film, a 15ml timing bolus of the contrast agent (Isovue 370 followed by a 20 ml saline chaser) was power injected and low dose scans of the base of skull area were taken every two seconds to determine time taken for the contrast to arrive at the internal carotid arteries at the skull base. The scan was discontinued when contrast appeared in these vessels and contrast arrival time was determined. The usual timeframe varied between 10–15 seconds and depended on patients cardiac output and placement of IV access.

### dCTA

**FIGURE 1.**
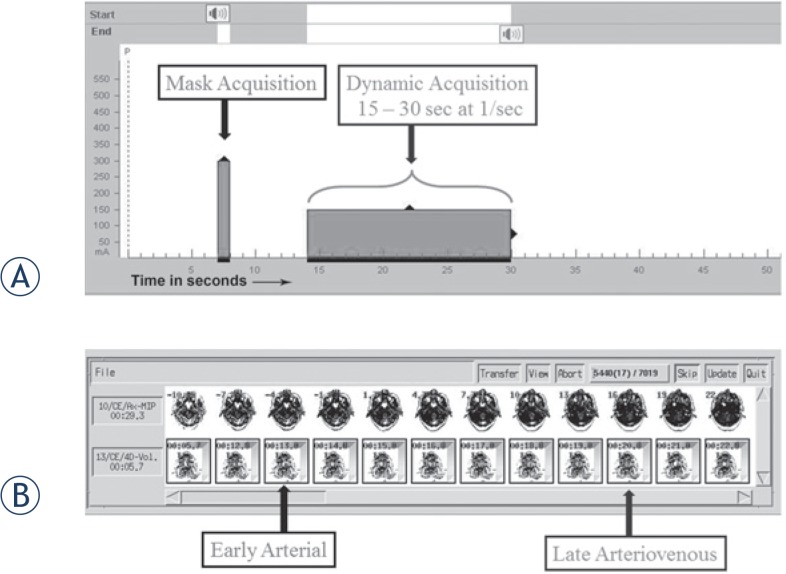
The upper panel **(A)** shows planning timeline for dCTA acquisition. Following simultaneous start of IV pump and the scanner, at 7 sec, a volume with 300 mA and 80 kV is taken as a mask for bone subtraction. This is a non-contrast image as the contrast bolus is yet to reach the cranial arteries. The next dynamic acquisition block (100 mA, 80 kV, 1 volume/sec for 16 volumes) will have different a start time, depending on the variable contrast arrival time at the internal carotid arteries at the base of skull as determined by the timing bolus. This will acquire 16 volumes starting 1 sec before the contrast arrival time. The lower panel **(B)** shows series of volumes following dCTA acquisition. Together they will show the timeline of contrast flow (dynamic CTA) thus permitting selection of the best volume showing the AVM nidus for CK planning.

**FIGURE 2.**
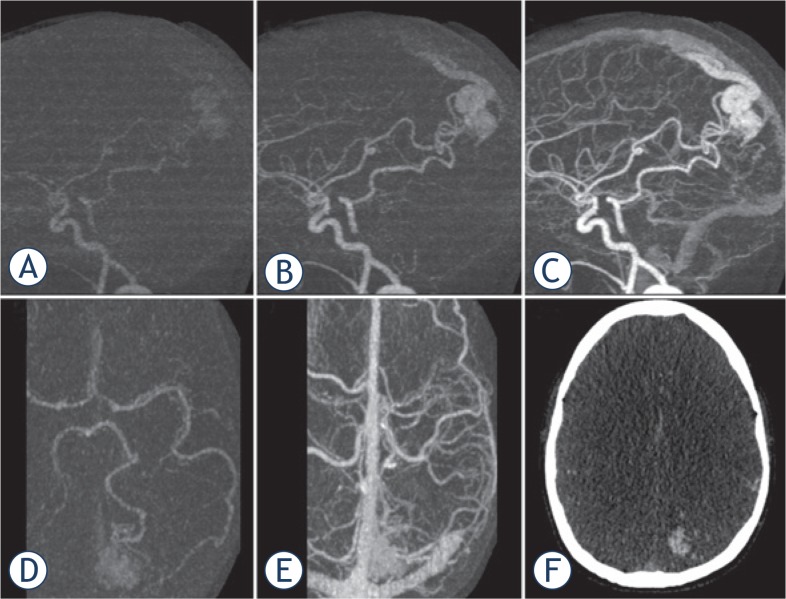
Sagittal reformatted dynamic subtracted images **(A, B, C)** show temporal flow of contrast through intracranial vessels and nidus of left occipital AVM. **(A)** very early arterial phase showing the AVM nidus before filling of contrast into the normal brain arteries due to rapid shunting through the AVM. Axial reformatted images in the arterial **(D)** and venous **(E)** phases demonstrate the difficulty in assessing the AVM nidus in the presence of enhancing surrounding vasculature. **(F)** an axial slice of non-subtracted dCTA volume co-registered in the CK system and used for SRS contouring.

The dCTA scan protocol (Figure 1) was initiated with simultaneous scanning and power injection of IV contrast (40 ml Isovue 370 @5ml/sec followed by a 20 ml saline chaser). A ‘mask’ scan was acquired at 7 seconds using 300mA and 80KV (Figure 1A). This dataset was used to digitally subtract bone from the angiogram datasets and was performed at higher mA to allow clearer definition of the intracranial vasculature. The scanner was setup for dynamic acquisition one second before the contrast arrived at the skull base (as determined from the timing bolus) at 100mA and 80 KV (Figure 1A). The whole brain volume from skull base to vertex was imaged once every second for 16 seconds and the complete data volume was reconstructed every 0.5 seconds (temporal resolution 2 images/sec). The duration of the scan allowed imaging to start with no contrast, continuing to the phase of peak arterial enhancement (Figure 2A) and ending with the venous return phase (Figure 2C).

Non-subtracted and bone subtracted dCTA datasets were created and reviewed by the neuroradiologist (SC) to determine the temporal phase of imaging where the AVM nidus was best visualised (Figure 1B and Figure 2F) without significant contrast in the draining veins and adjacent non-AVM vasculature. The optimum time-point for nidus visualisation varied between subjects depending on AVM flow rate.

### Treatment planning

The entire CT dataset (320 slices) at the time point where the nidus was clearest was reformatted to the CKRS treatment planning specifications (1x1x1mm cubic voxels) and imported as a DICOM file to the CK Multiplan TP system (Accuray, Sunnyvale CA). The dCTA was co-registered with the non-contrast planning CT and T2 weighted MRI to contour the target and the surrounding organs at risk. Coregistration between planning CT, dCTA and MRI was visually assessed in the standard split-screen manner using bone and ventricle landmarks. The 2D DSA was available for reference on an adjacent workstation. All imaging modalities were used to accurately delineate the nidus. Contouring was jointly performed by the neurosurgeon, radiation oncologist and neuroradiologist. Single fraction RS treatments were prescribed and the plan and the prescription isodose was finalized by the radiation oncologist to achieve coverage of the nidus and respect published normal tissue constraints.^12^

### Review of clinical use

Tolerability and apparent utility of the dCTA was assessed prospectively. Follow up imaging was performed 6 monthly using MRA, followed by a confirmatory catheter angiogram if the MRA indicated obliteration. Nidus obliteration rates and toxicity were evaluated from imaging and patient charts.

## Results

Between October 2010 and April 2012, 13 consecutive patients with inoperable AVMs were treated with CKRS at our hospital. Median age was 40 years (range 10–70). All patients tolerated the full dCTA protocol without adverse event and accurate co-registration of dCTA images with the CT and MRI within the CK planning system was performed in all cases.

Treatments were all delivered as a single fraction. Median marginal RS dose delivered was 16Gy (range 15–21Gy at the 75–85% isodose). Median target volume was 1.31cc (range 0.4–2.93cc). Ten patients were treated with RS because the AVM was in an eloquent or inoperable area, two patients as part of staged multimodality treatment following surgery and one patient because of medical comorbidities. Seven patients had prior intracranial haemorrhage. Two patients with thalamic AVMs had been previously treated at other centers with RS for AVM. Re-treatment in these cases occurred after a latency of five and fifteen years. Table 1 summarises the patient cohort and outcomes at a median follow-up of 37 months (range 28–45 months).

**FIGURE 3.**
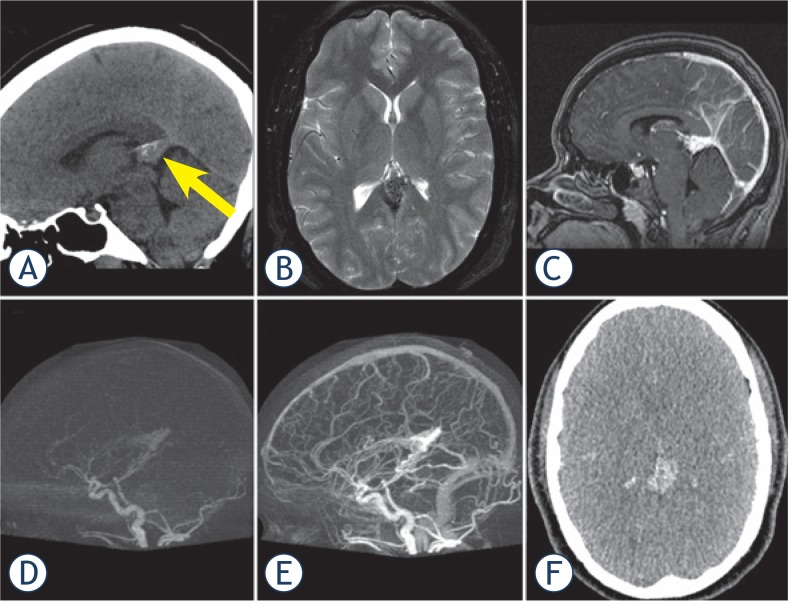
**(A)** Sagittal reformatted non-contrast CT head image showing iso-dense lesion in tentorial notch (arrow). **(B)** Axial T2 weighted MR image (3B) showing nidus with adjacent draining vein. **(C)** Sagittal reformatted image from T1 weighted VIBE image showing enhancing nidus with adjacent draining veins and venous sinuses. **(D–E)** Sagittal reformatted subtracted dCTA images in arterial **(D)** and venous **(E)** phase showing the AVM nidus in the arterial phase and occluded straight sinus. **(F)** Axial image from early arterial volume showing the nidus without contamination from surrounding enhancing vessels (full CT dataset from this temporal imaging phase imported into CBK for contouring).

**FIGURE 4.**
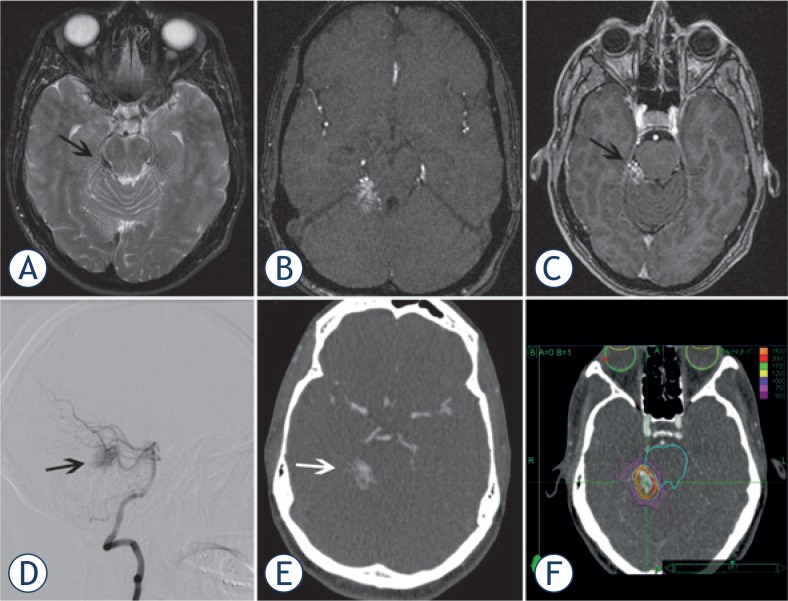
**(A)** Axial T2 image showing abnormal vasculature in right CP angle (black arrow). **(B)** Axial source image from the time of flight MR angiogram showing the nidus. **(C)** Source image from contrast enhanced axial T1weighted VIBE sequence showing enhancing AVM nidus with enhancement of adjacent vasculature. **(D)** arterial phase image of conventional catheter angiogram with injection from the left vertebral artery showing the AVM nidus (black arrow). This is a projectional 2-D image that cannot be coregistered in CBK. **(E)** sample axial image from the corresponding dCTA used for CK planning. **(F)** radiation target volume and isodose lines in CK planning system.

The RS team found that all imaging modalities were complementary. The dCTA data could be accurately co-registered within the CK Multiplan TPS and it was possible to visualize the nidus on both MRI and dCTA in all 3 planes. The sagittal and coronal images were compared to the reference 2D Angiography images in the adjacent work station. dCTA was felt by all three clinicians to be beneficial to help define the boundaries of the nidus in every case. On MRI alone it was often difficult to distinguish nidus from adjacent angiomatous change and draining veins often obscured the nidus on both MRI and on 2D Angiography. Careful selection of the optimal dCTA data set where there was minimal uptake in large draining veins and that excluded surrounding angiomatous change helped the RS team clarify boundaries of the nidus. In our experience the dCTA was also helpful to define portions of nidus extending into CSF space in periventricular AVMs and in cases where AVM nidus extended into adjacent sulci. Representative cases are illustrated in Figures 2–4.

### Example cases

#### Case 1 (patient 9)

A 36 year old female with a previous history of cervix cancer was investigated for headaches and found to have a 20 mm AVM in the left occipital area (Figure 2). The option of surgical management with risk of visual morbidity was discussed and patient declined. The patient had CKRS to a dose of 18 Gy to the 80% isodose and the nidus was completely obliterated at 22 months.

#### Case 2 (patient 10)

A 29 year old female investigated for headaches against a background of recent haemorrhagic stroke in a close relative, was found to have a 22 mm AVM in the pineal region (Figure 3). The AVM was located in a region deemed not amenable to surgery. The patient had CKRS to a dose of 16 Gy to the 85% isodose. The nidus was obliterated 28 months following treatment.

#### Case 3 (patient 11)

A 40 year old asymptomatic patient was found to have a 15 mm AVM in the right CP angle (Figure 4) when being screened for aneurysms. The patient received 15 Gy to the 77% isodose with CKRS with partial obliteration of the nidus after 22 months.

## Discussion

Accurate delineation of the AVM nidus is paramount to successful RS. The steep dose gradients delivered by RS mean that inaccurate targeting will result in a subtherapeutic dose to regions of the AVM and increase the likelihood of treatment failure. Conversely, treating a larger volume than required increases the risk of toxicity. However, it can be challenging to effectively distinguish the nidus from its feeding and draining vessels as reported by Buis *et al.*^13^, who evaluated intraobserver variability in contouring AVMs for RS. They reported a mean agreement ratio of 0.45 amongst six observers and showed that differences were most marked in those with treatment failure. Improved definition of the nidus through the use of multiple imaging modalities should increase the likelihood of successful AVM RS.

DSA remains the gold standard for visualisation of the nidus in view of the excellent spatial and temporal resolution, but the 2D nature of standard DSA limits its use in 3D RS contouring. Furthermore, it is not possible to coregister the DSA within CKRS planning software as CK is not frame-based and the DSA is therefore viewed on an adjacent imaging workstation. This introduces errors. Therefore centres generally co-register CTA or MRA to provide 3D cross-sectional data. However, even with timed contrast boluses and acquisition, the temporal resolution of standard CTA/MRA is limited to a snapshot view of the AVM. While this produces good cross-sectional imaging, the enlarged draining veins and contrast in adjacent non-AVM vasculature usually obscure parts of the nidus.

Several groups have reported the use of 3D reconstructions of DSA. Veeravagu *et al.*, reported smaller AVM target volumes for CKRS when contours were performed using a combination of 3D rotational angiography, CT and MRI versus CT and MRI alone.^14^ They concluded that the addition of 3D angiography into the planning protocol resulted in more precise target contouring, although patient outcome data and analysis of possible image distortion levels would strengthen their data. Zhang *et al.*, reported similar results several years earlier using in-house software to create a 3D reconstruction of DSA images.^15^ Columbo *et al.*, recently described a novel method of automatic nidus contouring using coregistered 3D rotational angiography (performed with direct intraarterial contrast injection).^16^ After establishing the radiological density of a region of nidus, the treatment planning software automatically contoured GTV based on corresponding voxel values, although manual correction was possible. They did not describe their validation process for this technique, but did report complete angiographic obliteration rates after ≥ three years in 65 of 80 patients (81.2%).

Various other non-invasive methods have been described that appear to assist in nidus definition in RS treatment planning or post-treatment follow-up. 3D time of flight (TOF) MRA has been advocated for the assessment of AVM nidus obliteration dynamics following CKRS^17^, although Bednarz *et al.*, concluded that DSA and TOF MRA were complementary for nidus RS delineation as imaging artefact on MRA could obscure the nidus.^18^ High sensitivity (81%) and specificity (100%) has been reported for dynamic MRA in the post-treatment evaluation of AVMs treated with RS.^19^

The use of dCTA for imaging AVMs was first described by Matsumo *et al.*^10^, who imaged four patients with AVM (within a study of various brain lesions) and reported that dCTA effectively distinguished the nidus from the feeding and draining vessels within the scanned range. Willems *et al.*, recently evaluated dCTA versus DSA to evaluate AVMs using a scoring system.^11^ They reported that dCTA could be used to effectively diagnose and classify the shunt but, in some circumstances there were discrepancies on classifying the angio-architecture with dCTA compared to DSA due to difficulty in determining the nature of certain vessels. However, they focused upon 3D maximum intensity projection (MIP) views of the dCTA in their comparison rather than the cross-sectional images we have used. Indeed, they went on to discuss the benefit of using the cross-sectional data to distinguish the nidus from surrounding vessels.

In this feasibility report we have shown that dCTA data can be easily imported into the CKRS planning system and co-registered to the planning CT and MRI. This allows contouring to be performed on 3D cross-sectional images of the nidus at the point when it is clearest. In all of our 13 cases it was possible to identify the optimum dCTA volume, directly import this 3D dataset into the CKRS software and accurately coregister with other modalities which allowed the nidus to be visualized in all 3 planes for contouring. The sagittal and coronal images can still be compared to the reference 2D angiography images on the adjacent work station. The AVMs in this report were of small volume which demands even greater accuracy than for larger lesions. Direct contouring on the dCTA on the CKRS planning software certainly was an advantage compared to our previous technique where contours were drawn on static CT/MRI images with reference to the DSA on an adjacent screen. It is important to carefully choose the right arterial phase of the dCTA and use the non-subtracted volume to allow confident definition of the nidus on the dCTA. However, at this point in our analysis it would be premature to conclude that dCTA replaces the need for DSA in RS planning and we found the combination of imaging modalities to be complementary.

The primary purpose of this study was to report our initial findings on the feasibility of using dCTA to assist in targeting AVMs for RS on the Cyberknife TPS. Accordingly, our data is descriptive. We do not claim to have validated dCTA in nidus contouring. Formal validation of a new imaging technique in contouring is challenging. Although comparison of volumes contoured with and without the dCTA may show differences, such differences alone do not themselves reflect whether the dCTA improves contouring accuracy. Improvement in contouring agreement between observers using a new imaging technique is often used as a surrogate for improved accuracy. However, we did not pursue this approach as we contour as a multidisciplinary group with different areas of primary expertise (radiology, neurosurgery and radiation oncology) and our final targets reflect a consensus opinion. Long-term patient outcomes will be the ultimate validation, but patient numbers are too low and follow-up too short to accurately assess treatment success rates at this point as the latency period to complete obliteration following RS can be 4–5 years. We did evaluate preliminary patient outcomes to establish whether it is reasonable to continue to study dCTA in AVM RS treatment planning and the data available so far is comparable to other published outcomes at this follow-up period. Complete nidus obliteration rates three years post-RS of 58%, 39% and 40% have been reported by various authors^7^,^20^,^21^, with successful obliteration rates increasing as follow-up continues. Six of our first thirteen patients (46%) contoured using dCTA had complete nidus obliteration at a median follow-up of 37 months. Five other patients have had partial responses and the nidus size continues to progressively decrease suggesting they may completely obliterate with further follow-up. Due to the small nidus volume in our cohort, we would hope for complete obliterations in approximately 80% of patients after 4–5 years follow-up. Clearly we do not suggest that our current outcomes themselves validate dCTA in contouring at this point, but we continue to prospectively collect long-term patient outcome and toxicity data. Two patients, both with complete nidus obliterations, required delayed steroid therapy, one for simple edema and one for a thrombosed draining vein, an unusual but documented potential complication of RS for AVM.^22^ Review of this patients RS contours showed that the addition of the dCTA had in fact reduced the volume of draining vein included in the target.

## Conclusions

It is feasible to integrate dCTA into a CKRS planning protocol for AVM delineation. This technique combines the better spatial resolution of 3D CT volumes with the ability to select the best temporal phase of contrast filling. In our preliminary evaluation, we found dCTA to be a complementary addition to the other standard imaging modalities used to contour the AVM nidus and particularly useful for CK planning as DSAs cannot be imported into the CK TPS. We have now incorporated dCTA into our standard treatment planning protocol and will continue to prospectively collect longer-term follow-up data in more patients for validation.

## Figures and Tables

**TABLE 1.** Patient characteristics and outcomes

**Pt**	**Region**	**Age**	**Dose Gy**	**Isodose Tx (%)**	**Prev bleed**	**Prev RT**	**Success?**	**Comments**	**Follow-up (months)**
**1**	L basal ganglia	10	15	84	Yes	No	N	Embolization after 33 months	45
**2**	L thalamus	40	15	82	Yes	Yes	N	RS 15 years previous (lost to follow-up)	45
**3**	R occipital	50	18	85	No	No	C		45
**4**	L cerebellum	49	15	84	Yes	No	P	Large AVM – only deep nidus treated, for embolization of remainder	44
**5**	L vein of galen	22	15	80	Yes	No	P		44
**6**	R parietal	70	18	82	No	No	C		38
**7**	Corpus callosum	22	16.5	75	No	No	C		37
**8**	Sup cerebellum	61	20	80	Yes	No	C		34
**9**	L occipital AVM	36	18	80	No	No	C		32
**10**	Pineal	29	16	85	No	No	C		30
**11**	Sup cerebellum	58	21	82	Yes	No	P		30
**12**	R CP angle	40	15	77	No	No	P		29
**13**	R thalamus	11	15	78	Yes	Yes	P	RS 5 years previously	28

C = complete obliteration; L/R = left and right; N = no obliteration; P = partial obliteration; Pt = patient
