# Erratum

**DOI:** 10.1515/raon-2015-0029

**Published:** 2015-08-21

doi: 10.1515/raon-2015-0006

The corresponding author should be Santanu Chakraborty instead of Jillian Mclean.

The corresponding author should be:

Dr. Santanu Chakraborty, Department of Radiology, University of Ottawa, Department of Medical Imaging, The Ottawa Hospital, 1053 Carling Avenue, Ottawa, ON K1Y 4E9, Canada. Phone: (613) 737 8571; Fax: (613) 761 4476; E-mail: schakraborty@toh.on.ca

